# Effects of acidosis on neuronal voltage-gated sodium channels: Nav1.1 and Nav1.3

**DOI:** 10.1080/19336950.2018.1539611

**Published:** 2018-10-26

**Authors:** Mohammad-Reza Ghovanloo, Colin H. Peters, Peter C. Ruben

**Affiliations:** Department of Biomedical Physiology and Kinesiology, Simon Fraser University, Burnaby, Canada

**Keywords:** Acidosis, electrophysiology, Nav1.1, Nav1.3, pH

## Abstract

Voltage-gated sodium channels are key contributors to membrane excitability. These channels are expressed in a tissue-specific manner. Mutations and modulation of these channels underlie various physiological and pathophysiological manifestations. The effects of changes in extracellular pH on channel gating have been studied on several sodium channel subtypes. Among these, Nav1.5 is the most pH-sensitive channel, with Nav1.2 and Nav1.4 being mostly pH-resistant channels. However, pH effects have not been characterized on other sodium channel subtypes. In this study, we sought to determine whether Nav1.1 and Nav1.3 display resistance or sensitivity to changes in extracellular pH. These two sodium channel subtypes are predominantly found in inhibitory neurons. The expression of these channels highly depends on age and the developmental stage of neurons, with Nav1.3 being found mostly in neonatal neurons, and Nav1.1 being found in adult neurons. Our present results indicate that, during extracellular acidosis, both channels show a depolarization in the voltage-dependence of activation and moderate reduction in current density. Voltage-dependence of steady-state fast inactivation and recovery from fast inactivation were unchanged. We conclude that Nav1.1 and Nav1.3 have similar pH-sensitivities.

## Introduction

Electrical signalling is a vital part of biology in many organisms. A key component of this signalling depends on a rapid, transient, and all-or-none process known as the action potential. Action potentials are generated and propagated through various ion channels, including voltage-gated sodium channels (Nav) [1]. The sodium current passing through these channels initiates action potentials in neurons, and skeletal and cardiac muscles. Nav channels are hetero-multimeric proteins composed of large, ion conducting α-subunits and smaller auxiliary β-subunits [1–7]. The α-subunit is made up of a single gene transcript that encodes four 6-transmembrane segment domains [1]. Each one of these four structural domains can be divided by function into the voltage-sensing domain (VSD) and the pore domain (PD) [1,2]. These two functional domains are connected through the intracellular S4-S5 linker [1,8]. The VSD is formed by the first four transmembrane segments of each domain and the PD is formed by the 5^th^ and 6^th^ segments along with the extracellular pore loop that connects them [1,2].

**Figure 1.**
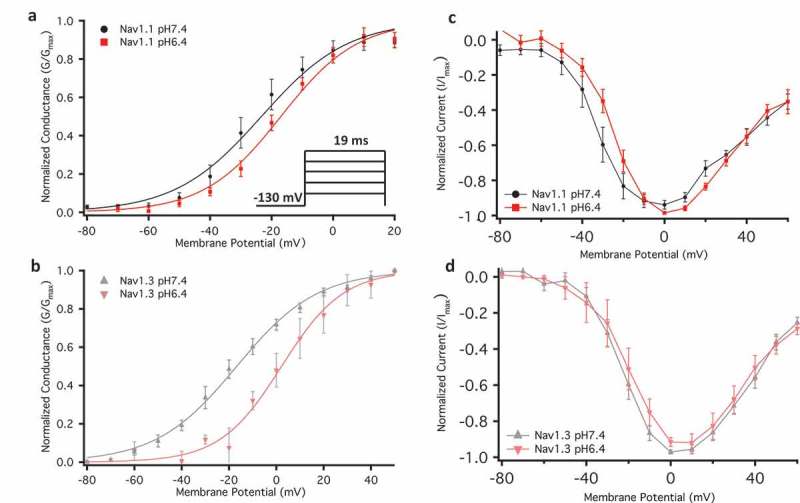
Normalized conductance plotted against membrane potential. (a-b) Show overlaps of Nav1.1 and Nav1.3 conductance at pH6.4 and 7.4. The inset in panel (a) shows voltage protocol used. (c-d) Normalized current and voltage relationships.

**Figure 2.**
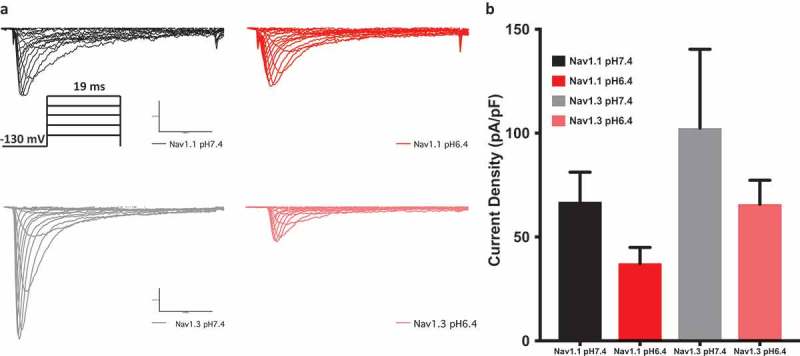
Current density measured in pA/pF. (a) Sample macroscopic sodium currents elicited by depolarizations between −100 and + 80 mV. The inset in panel (a) shows voltage protocol used. (b) Average current (Y-axis) density of Nav1.1 and Nav1.3 at extracellular pH between 6.4 and 7.4.

**Figure 3.**
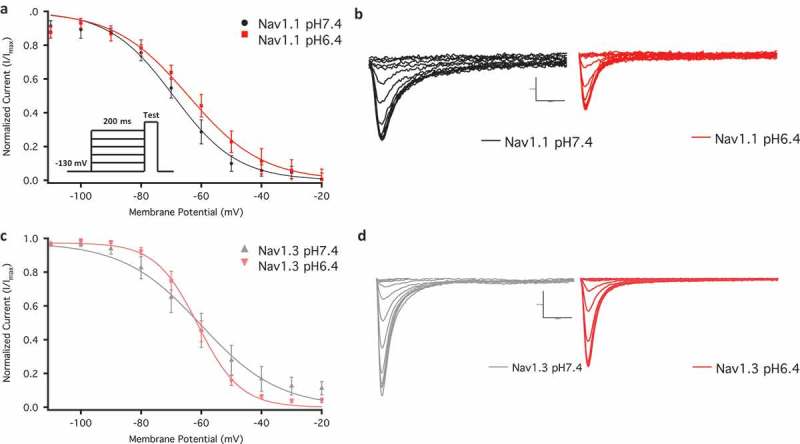
Voltage-dependence of steady-state fast inactivation as normalized current plotted against membrane potential. (a) Show the voltage-dependence of fast inactivation of Nav1.1 at pH6.4 and pH7.4. The inset shows voltage protocol used. (b) Inactivating current traces associated with Nav1.1 in pH6.4 and pH7.4. (c) Show the voltage-dependence of fast inactivation of Nav1.3 at pH6.4 and pH7.4. (d) Inactivating current traces associated with Nav1.3 in pH6.4 and pH7.4.

**Figure 4.**
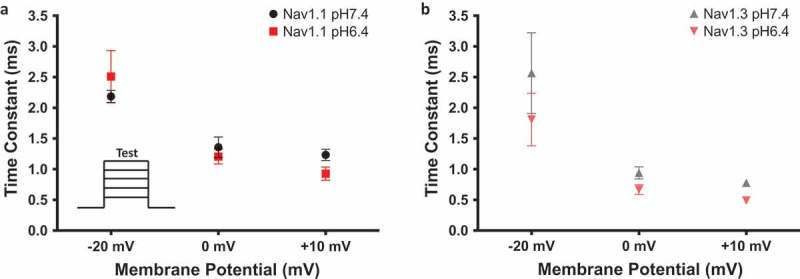
Open-state fast inactivation time constants. (a-b) Time constants at −20, 0 and + 10 mV from Nav1.1 and Nav1.3 at pH6.4 and pH7.4. The inset in (a) shows the voltage protocol that was used.

**Figure 5.**
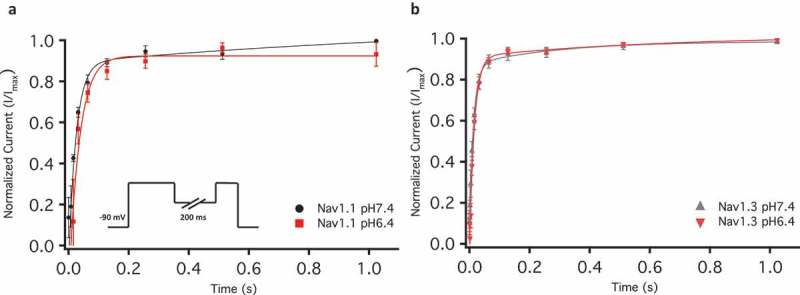
Recovery from fast inactivation. (a-b) Show the normalized current is plotted against a range of recovery durations (s). The inset in (a) shows the pulse protocol that was used.

**Figure 6.**
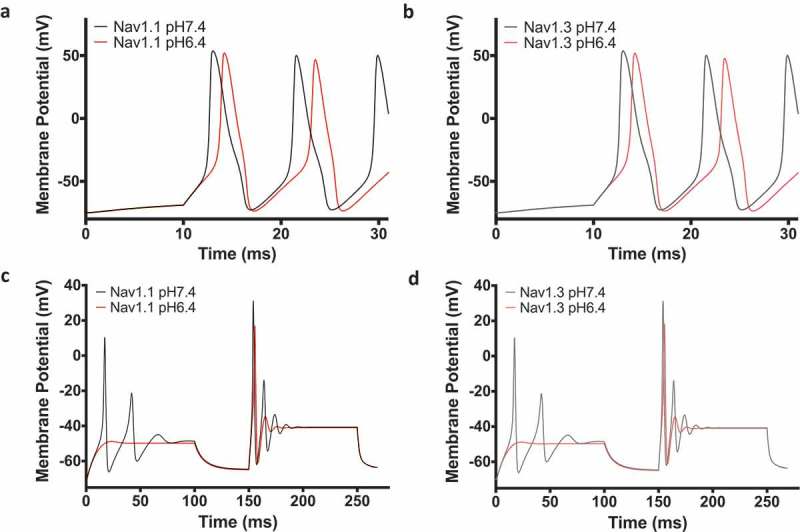
Action potential model. (a-b) Threshold level action potential simulation of Nav1.1 and Nav1.3 at pH7.4 and pH6.4. (c-d) Action potential simulations at increasing and sustained current injection intensities.

In a simplified model, the sodium channel can exist in three fundamental states: resting, open, and inactivated [1]. During depolarizations that are of sufficient magnitude, sodium channels activate (outward movement of VSD), enter the open-state (opening of PD), and begin conducting ionic sodium currents [8]. Any disturbance to this process of sodium current conduction could have downstream effects on excitability. After activation, sodium channels may enter a fast-inactivated state, which is initiated by the outward movement of the domain IV-VSD. Fast inactivation is mediated by the domain III-IV linker, known to be the fast inactivation gate, binding to the inside of the channel pore [9]. This process that happens within milliseconds of activation, blocks the channel pore, and effectively stops current conduction. Thus inactivation is a way of regulating excitability. Inactivation can proceed from either closed or open states of the channel, which are known as “closed-state inactivation” and “open-state inactivation”, respectively [10,11].

There are multiple sodium channel isoforms expressed in tissues throughout the body. Nav1.1-Nav1.3 are found in the central nervous system. Nav1.4 and Nav1.5 are expressed in skeletal and cardiac muscles, respectively. Nav1.6 is expressed in both the central and peripheral nervous systems. Nav1.7-Nav1.9 are primarily found in the peripheral nervous system. The expression pattern of the neuronal Nav channels depends on both the developmental stage, brain region, and cell type. Nav1.3 is expressed predominantly in neonatal brain cells; thus, it is believed to be a key contributor to brain development. In contrast, Nav1.1, Nav1.2, and Nav1.6 are highly expressed in adult brains. Furthermore, Nav1.2 displays greatest expression in unmyelinated axons, whereas Nav1.6 is found in the cell soma [12,13]. Although the different isoforms share a similar structure, their gating and response to physiological and pathophysiological modulators can vary widely. In particular, sodium channels vary in their response to changes in extracellular pH, with Nav1.5 being more sensitive than Nav1.2 or Nav1.4.

Maintaining the physiological pH balance is of vital importance to human body function. Under normal physiological conditions, the extracellular pH is maintained at approximately 7.4, with the intracellular pH ranging between 7.2 and 7.4. Under hypoxic and ischemic conditions, the extracellular pH decreases significantly. During focal ischemia, rabbit brain pH could drop to 6.0 (brain intracellular pH is 7.0) [14]. Similarly, intense physical exercise has been shown to result in extracellular pH in muscle tissue decreasing to 6.4 [15,16]. In cardiac tissue, during myocardial ischemia, including regional and global ischemia, extracellular pH can lower to 6.1 [17]. The presence of extracellular protons can modulate both the VSD and the PD, depolarizing the voltage-dependence of activation and blocking ionic current, respectively [18,19]. Acidification decreases peak sodium conductance by protonating the outer vestibule carboxylates [19,20], and likely by binding to negative charges in the VSDs, which destabilizes the outward conformation of the voltage-sensors [21,22].

Recently, our group has shown that a mutation (P1158S) that alters the structure of the S4-S5 linker could increase pH-sensitivity in the otherwise pH-insensitive Nav1.4. The pathological consequences of pH modulation of sodium channel mutants with an increased sensitivity to extracellular protons, E1784K (Nav1.5) and P1158S (Nav1.4), include Brugada syndrome, long QT syndrome, periodic paralysis, and myotonia [23–25].

We previously reported that Nav1.2 and Nav1.4 display relative insensitivity to protons compared to Nav1.5 [24,26,27]. However, little is known about proton effects in most sodium channels, including the neuronal subtypes. For instance, only one study has investigated the effects of protonation in Nav1.1, showing that protons block current and depolarize activation [28]. To investigate the effects of acidosis on the gating properties of Nav1.1 and Nav1.3, we performed whole-cell patch-clamp experiments [29]. Our findings suggest that, consistent with the similar distribution of Nav1.1 and Nav1.3 in neurons, these channels display an almost identical level of pH-sensitivity. At low pH, both channels displayed a depolarizing shift in their voltage-dependence of activation; however, neither channel showed a significant shift in their voltage-dependence of inactivation or recovery from inactivation, and both showed an accelerated open-state inactivation only at highly depolarized potentials.

## Methods

### Cell culture

Chinese Hamster Ovary (CHO) cells were transiently co-transfected with cDNA encoding eGFP and the β1-subunit and either the Nav1.1 or Nav1.3 α-subunit. Transfection was done according to the PolyFect transfection protocol. After each set of transfections, a minimum of 8-hour incubation was allowed before plating on sterile coverslips.

### Electrophysiology

Whole-cell patch-clamp recordings were performed in an extracellular solution containing (in mM): 140 NaCl, 4 KCl, 2 CaCl_2_, 1 MgCl_2_, 10 HEPES (pH 7.4) or MES (pH 6.4). Solutions were adjusted to pH (6.4, 7.4) with CsOH. Pipettes were filled with intracellular solution, containing (in mM): 120 CsF, 20 CsCl, 10 NaCl, 10 HEPES. All recordings were made using an EPC-9 patch-clamp amplifier (HEKA Elektronik, Lambrecht, Germany) digitized at 20 kHz via an ITC-16 interface (Instrutech, Great Neck, NY, USA). Voltage-clamping and data acquisition were controlled using PatchMaster software (HEKA Elektronik, Lambrecht, Germany) running on an Apple iMac. Current was low-pass-filtered at 10 kHz. Leak subtraction was performed automatically by software using a P/4 procedure following the test pulse. Gigaohm seals were allowed to stabilize in the on-cell configuration for 1 min prior to establishing the whole-cell configuration. Series resistance was less than 5 MΩ for all recordings. Series resistance compensation up to 80% was used when necessary. All data were acquired at least 1 min after attaining the whole-cell configuration. Before each protocol, the membrane potential was hyperpolarized to −130 mV to ensure complete removal of both fast inactivation and slow inactivation. All experiments were conducted at 22 °C.

### Activation protocols

To determine the voltage-dependence of activation, we measured the peak current amplitude at test pulse potentials ranging from −100 mV to + 80 mV in increments of + 10 mV for 20 ms. Channel conductance (G) was calculated from peak I_Na_:

(1) $$G_{Na}=I_{Na}/V-E_{Na}$$

where G_Na_ is conductance, I_Na_ is peak sodium current in response to the command potential V, and E_Na_ is the Nernst equilibrium potential. Calculated values for conductance were fit with the Boltzmann equation:

(2) $$G/G_{max}=1/\left(1+exp[-ze_{0}[V_{m}-V_{1/2}\right]/kT])$$

where G/G_max_ is normalized conductance amplitude, V_m_ is the command potential, z is the apparent valence, e_0_ is the elementary charge, V_1/2_ is the midpoint voltage, k is the Boltzmann constant, and T is temperature in K.

### Steady-state fast inactivation protocols

The voltage-dependence of fast inactivation was measured by preconditioning the channels to a hyperpolarizing potential of −130 mV and then eliciting pre-pulse potentials that ranged from −170 to + 10 mV in increments of 10 mV for 500 ms, followed by a 10 ms test pulse during which the voltage was stepped to 0 mV. Normalized current amplitudes from the test pulse were fit as a function of voltage using the Boltzmann equation:

(3) $$I/I_{max}=1/(1+exp\left(-ze_{0}\left(V_{M}-V_{1/2}\right)/kT\right)$$

where *I*_max_ is the maximum test pulse current amplitude.

### Open-state fast inactivation measurements

We measured open-state fast inactivation by fitting the decay of macroscopic currents with a single exponential function. This was measured at −20, 0, and + 10 mV.

### Recovery from fast inactivation protocols

Channels were fast-inactivated during a 20 ms or 200 ms depolarizing step to 0 mV, and recovery was measured during a 19 ms test pulse to 0 mV following a −90 mV recovery pulse for durations between 0 and 1.024 s. Time constants of fast inactivation recovery showed two components and were fit using a double exponential equation:

(4) $$I=Iss+\alpha 1exp\left(-t/\tau 1\right)+\alpha 2exp\left(-t/\tau 2\right)$$

where *I* is current amplitude, *I*_ss_ is the plateau amplitude, α_1_ and α_2_ are the amplitudes at time 0 for time constants τ_1_ and τ_2_, and *t* is time.

### Action potential modeling

Neuronal action potential modeling was based on a modified Hodgkin-Huxley model [30]. The equations in the model were modified to reflect the properties of cortical pyramidal cells [31,32].

In our simulations, the pH 7.4 parameters were matched to those in cortical pyramidal cells (original model), and the pH 6.4 parameters were shifted based on electrophysiological results obtained from whole-cell patch-clamp experiments in this study. Only sodium current parameters were changed, leaving open the question as to the effects of extracellular acidification on other channel types (e.g. potassium channels). The model accounted for activation voltage-dependence, steady-state fast-inactivation voltage-dependence, and peak sodium currents; however, only the statistically significant parameters (voltage-dependence of activation) were changed relative to the original model parameters. The program was coded in the Python language.

### Analysis

Analysis and graphing were done using FitMaster software (HEKA Elektronik) and Igor Pro (Wavemetrics, Lake Oswego, OR, USA). All data acquisition and analysis programs were run on an Apple iMac (Apple Computer). Statistical analysis was performed in JMP version 13.

### Statistics

A t-test was used to compare the mean responses [activation, current density, steady-state fast inactivation, open-state fast inactivation, and fast inactivation recovery] between the two pH points in each channel variant. pH had two levels (pH 6.4 and pH 7.4). A level of significance α = 0.05 was used in all overall tests, and effects with p-values less than 0.05 were considered to be statistically significant. All values reported are given as means ± standard error of means for n cells.

## Results

### Low pH destabilizes activation in Nav1.1 & Nav1.3

We examined the effects of pH changes on activation by measuring peak channel current and determining conductance (see Eq. 1) at membrane potentials between −100 and + 80 mV (Figure 1(a-d)). We found that decreasing the extracellular pH causes significant shifts on the midpoint of the conductance curve (V_1/2_) of both channels (Nav1.1: p = 0.0046, Nav1.3: p = 0.0037) in the depolarized direction; however, the apparent valence (z) of activation was unchanged in both channels during acidosis (p > 0.05) (Figure 1(a,b); Table 1). This suggests that although acidosis has a destabilizing effect on the voltage-dependence of activation, it does not affect the magnitude of charge movement during activation.

**Table 1.** Conductance.

Channel Type	Mean V_1/2_ ± SE (mV)	Mean z ± SE (slope)	n
Nav1.1 pH6.4	−18.9 ± 2.0	2.5 ± 0.2	7
Nav1.1 pH7.4	−27.8 ± 2.0	2.7 ± 0.2	7
Nav1.3 pH6.4	−7.3 ± 2.4	3.0 ± 0.2	5
Nav1.3 pH7.4	−16.5 ± 1.6	2.8 ± 0.2	11

### Protons moderately decrease peak current density in NaV1.1 & Nav1.3

Previous studies show that an increased concentration of positively charged H^+^ during acidosis results in the protonation of the carboxylates of the outer channel vestibule [19,25]. Although this protonation is not the only determinant of proton block, it is a key component [19,25,33,34]. To determine the extent of proton block in Nav1.1 and Nav1.3, we measured current density from the ratio of peak current amplitude to the cell membrane capacitance (pA/pF) at pH 6.4 and pH 7.4. Representative traces of macroscopic families of currents are shown in (Fig. 2A). Although the current densities of both Nav1.1 and Nav1.3 channels were decreased at the lower pH, these decreases were not statistically significant (p > 0.05) (Figure 2(b); Table 2). This moderate reduction of peak current amplitude is similar to that seen in Nav1.4 [24].

**Table 2.** Current density.

Channel Type	Mean density ± SE (pA/pF)	n
Nav1.1 pH6.4	37.3 ± 19.3	7
Nav1.1 pH7.4	67.0 ± 18.1	8
Nav1.3 pH6.4	65.9 ± 19.3	7
Nav1.3 pH7.4	102.4 ± 20.9	6

### Acidosis has no effects on steady-state fast inactivation

We measured the voltage-dependence of steady-state fast inactivation using a standard pre-pulse voltage protocol. Normalized current amplitudes were plotted as a function of pre-pulse voltage (Figure 3(a-d)). Our results indicate that acidosis does not significantly alter either the V_1/2_ or z of the voltage-dependence of steady-state fast inactivation in Nav1.1 or Nav1.3 (Figure 3(a-d); Table 3) (p > 0.05). This lack of impact on the voltage-dependence of fast inactivation is similar to that observed in Nav1.2 and Nav1.4 [24,26]. We show representative current traces of inactivating channels in both channels across conditions in (Figure 3(c,d)).

**Table 3.** Steady-state fast inactivation.

Channel Type	Mean V_1/2_ ± SE (mV)	Mean z ± SE (slope)	n
Nav1.1 pH6.4	−64.3 ± 3.0	2.0 ± 0.3	8
Nav1.1 pH7.4	−69.1 ± 3.0	2.2 ± 0.3	8
Nav1.3 pH6.4	−61.9 ± 3.5	3.8 ± 0.3	6
Nav1.3 pH7.4	−63.5 ± 3.5	2.9 ± 0.3	6

### Low ph accelerates onset of NaV1.1 & Nav1.3 open-state inactivation but not recovery

Our previous results in Nav1.2 and Nav1.5 show that the onset of inactivation is slowed during acidosis [27,35]. In the present study, we measured the open-state inactivation time constants associated with Nav1.1 and Nav1.3 at three membrane potentials of: −20, 0, and + 10 mV. Our results suggest that these time constants become smaller at pH 6.4 than pH 7.4 at more depolarized membrane potentials. This change in time constants was statistically significant at + 10 mV (Figure 4(a,b)) (Nav1.1: p = 0.0149, Nav1.3 = 0.0274). This finding contrasts with previous reports in Nav1.2 and Nav1.5, and suggests that the protonation of domain IV occurs to a lesser extent in Nav1.1 and Nav1.3 [35,36]. Similarly, the recovery from inactivation in Nav1.1 and Nav1.3 is not affected by acidosis (Figure 5(a,b); Table 4) (p > 0.05). This result also contrasts with Nav1.2 and Nav1.5 and is consistent with the idea of having fewer proton-channel interaction sites at domain IV in Nav1.1 and Nav1.3.

**Table 4.** Fast inactivation recovery.

Channel Type	Mean ± SE (s)	n
Nav1.1 pH6.4	0.027 ± 0.005	5
Nav1.1 pH7.4	0.020 ± 0.005	6
Nav1.3 pH6.4	0.015 ± 0.004	7
Nav1.3 pH7.4	0.010 ± 0.005	6

### Low pH reduces neuronal excitability in a Hodgkin & Huxley model of a neuron

To test the effects of low pH on neuronal excitability, we used a Hodgkin & Huxley-based model to simulate action potentials [30–32]. The physiological pH conditions were simulated using the original model parameters, and the low-pH action potentials were simulated using modifications to the original parameters that were based on our experimental data. Threshold level simulations suggest that, at low pH, the action potential upstroke is delayed (Figure 6(a-b)). Consistent with the similarities in the depolarizing shifts in activation in Nav1.1 and Nav1.3, the simulation of action potential morphology is also similar. In the next set of simulations, the channels were given two series of step-wise current injections with increasing intensities at each step for 100 ms. The first 100 ms injection interval was followed by a 50 ms recovery period in which no current injection was applied. Our results from these simulations suggest that low pH reduces neuronal excitability. This is shown by a reduced number of action potentials, and shortened amplitude of spikes during the second injection interval (Figure 6(c,d)).

## Discussion

There are many physiological and pathophysiological events that alter blood pH levels. These pH changes can impact the normal function of various protein systems which, in turn, could lead to clinical conditions. Therefore, the human body has evolved several mechanisms to maintain this crucial acid-base homeostasis, such as the renal system [15]. Electrical excitability is among the physiological systems affected by acidosis [26,37]. The results of our study demonstrate additional mechanisms by which neuronal excitability is altered by extracellular acidosis. Our results suggest that, in neurons expressing Nav1.1 or Nav1.3, excitability could be decreased during extracellular acidification.

Previous studies in sodium channels determined that mutations in the conserved DEKA and EEDD motifs cause a shift in the pKa of proton block in the acidic direction [19,33,34]. Mutating these carboxylates into alanine residues results in an approximately 25% decrease in proton block. This suggests that the interactions between the positively charged H^+^ and pore carboxylates blocks the ion conductance pathway, which subsequently reduces sodium current [34]. As DEKA and EEDD are conserved across the sodium channel family, it is not surprising that, in all of the sodium channels studied thus far, proton block exists albeit with varying degrees [25,26]. This is particularly evident in Nav1.4 where previous studies have shown a reduction in current density that is neither statistically significant nor negligible [26,38].

Proton block is not limited to the protonation of carboxylates in the selectivity filter. Previous studies in Nav1.5 identified a cysteine (C373) residue on the outer vestibule of domain I that imparts pH-sensitivity [19,20]. In Nav1.1–1.4 this cysteine is replaced with either phenylalanine or tyrosine residues. During acidosis, C373 gets protonated, creating a positive charge outside the pore that causes proton block. The presence of this cysteine can in part explain the increased pH-sensitivity observed in Nav1.5 compared to other sodium channels [18].

In addition to C373, two other residues involved in pH response were identified in Nav1.4 and Nav1.5. H880 in Nav1.5 is located in the pore loop of domain II, and P1158 in Nav1.4 is located on the hinge of the intracellular S4-S5 linker of domain III. Both these residues are conserved in Nav1.1-Nav1.5 [18,24] and are important to the biophysical properties of the respective channels in which they were described. Mutating H880 into a glutamine (Q) residue reduces the pH-sensitive current and shifts the voltage-dependence of activation in Nav1.5. Unlike C373 and H880, both of which directly contribute to proton block in Nav1.5 [18], P1158 in Nav1.4 indirectly contributes to a reduced proton-insensitivity. P1158 is located on the intracellular side of the channel, and we previously showed that mutating this proline to a serine (S) residue increases proton block in Nav1.4 at low pH [24]. This effect may occur by altering the voltage-dependence of gating in domain III.

In addition to proton block, low pH alters channel gating. The effects of protons on gating have been thoroughly studied in Nav1.5 [25]. Although the identity of the residues involved in pH-dependent changes in gating have not been fully determined, structural studies in bacterial sodium channels and potassium channels suggest that acidic residues play a role [21,39]. Interactions of protons at the individual domains typically depolarizes the voltage-dependence, presumably via electrostatic interactions which hinder the outward movement of S4 voltage-sensors. This electrostatic hindrance at domains I-III primarily affects activation, and at domain IV affects fast inactivation. However, the effects of protons on sodium channel gating is strongly subtype-dependent [24–26],(Table 5).

**Table 5.** Response to acidosis among sodium channels.

Subtype	Activation Relative to pH7.4	Inactivation Relative to pH7.4	Reference
Nav1.1	Depolarized	Unchanged	This study
Nav1.2	Depolarized	Unchanged	[26,27]
Nav1.3	Depolarized	Unchanged	This study
Nav1.4	Unchanged	Unchanged	[24,26]
Nav1.5	Depolarized	Depolarized	[35]

In this study, we characterized the effects of protons on Nav1.1 and Nav1.3. Our results suggest that the magnitude of the proton-dependent changes in the biophysical properties of these channels is nearly identical. This similarity is consistent with the shared localization of Nav1.1 and Nav1.3 in the cell bodies of neurons, which may suggest similar roles in neuronal excitability, as the expression of these channels are inversely correlated during neonatal development and postnatal weeks [12,40–43]. We found that at low pH, both channel subtypes display a depolarized conductance-voltage relationship, but no effects on steady-state fast inactivation. These results are consistent with the pH-sensitivity reported for rat pyramidal neurons [37].

The comparison of proton-sensitivity across Nav1.1-Nav1.5 reveals that activation is more susceptible to pH modulation than inactivation (Table 5). There are two potential explanations for this observation that are not mutually exclusive: 1) there is more exposure to extracellular protonation of sites involved in activation, and 2) having one domain controlling fast inactivation instead of three controlling activation decreases the number of protonatable sites and therefore decreases the probability that protons modulate fast inactivation. Testing the first hypothesis requires extensive mutation-based experimentation that should be investigated in future studies. The second hypothesis is based on the classic Hodgkin-Huxley model that describes the sodium conductance in terms of three activation components and a single fast inactivation component (*g_Na_ = m^3^h*) [30]. Thus, it is conceivable that having more domains controlling activation may increase the likelihood of carboxylate-proton interactions in domains I-III, which is in part due to having a larger net number of carboxylate-containing residues to protonate.

Although much effort has gone into gaining insight into pH-sensitivity of sodium channels, many questions remain unanswered. In this study, our goal was to determine the nature and extent of previously untested proton-sensitivity in Nav1.1 and Nav1.3. We observed nearly identical pH responses in Nav1.1 and Nav1.3, and significant differences between these channel subtypes and Nav1.2. Our results further elucidate the exquisite complexity of proton-sensitivity in sodium channels. This complexity needs to be explored further in future studies.
